# Perceived support from AI health tools and exercise adherence among university students: a three-wave study of exercise self-efficacy and autonomous motivation

**DOI:** 10.3389/fpsyg.2026.1872095

**Published:** 2026-10-12

**Authors:** Yuchao Lu, Ruihong Tang, Baijie Zeng, Jiazeng Sun, Shaoheng Liang, Degui Fu

**Affiliations:** 1 The Affiliated Guangdong Second Provincial General Hospital of Jinan University, Guangzhou, China; 2 Hunan First Normal University, Changsha, China; 3 Department of Educational Psychology, University of Wisconsin-Milwaukee, Milwaukee, WI, United States; 4 Guangdong Pharmaceutical University, Guangzhou, China

**Keywords:** artificial intelligence health tools, exercise adherence, exercise self-efficacy, perceived exercise support, university students

## Abstract

Maintaining regular exercise is challenging for many university students, and AI-enabled health tools are increasingly used to provide individualized exercise guidance and support. This study examined whether perceived exercise support from AI health tools was prospectively associated with later exercise adherence and whether exercise self-efficacy and autonomous motivation were involved in this association. A three-wave survey was conducted among 438 university students in China who were existing users of AI health tools with exercise-support functions. Perceived AI exercise support and baseline exercise adherence were assessed at T1, exercise self-efficacy at T2, and autonomous motivation and exercise adherence at T3. Structural equation modeling with bias-corrected bootstrap procedures was used, with baseline exercise adherence and relevant covariates controlled. Perceived AI exercise support at T1 was positively associated with exercise self-efficacy at T2 (β =0.36, *p* <0.001), which was positively associated with autonomous motivation at T3 (β =0.41, *p* <0.001). Autonomous motivation at T3 was concurrently associated with exercise adherence at T3 (β =0.29, *p* <0.001). The serial indirect association was significant (β = 0.04, 95% CI [0.02, 0.06]), and the direct association between perceived AI exercise support and T3 exercise adherence remained significant (β =0.11, *p* = 0.028). These findings are consistent with a psychological sequence in which perceived AI exercise support is associated with greater exercise self-efficacy and more autonomous motivation, which in turn is associated with exercise adherence. Because autonomous motivation and adherence were measured concurrently at T3, the final link should be interpreted as a within-wave association rather than as evidence of a fully longitudinal causal mediation process.

## 1 Introduction

Regular physical activity is widely recognized as an important contributor to physical and psychological wellbeing during emerging adulthood. Physical activity may also help reduce repetitive negative thinking (Wang et al., 2025c). However, for university students, the challenge often lies not simply in initiating exercise, but in maintaining exercise adherence over time. Compared with earlier educational stages, university life is characterized by less structured routines, greater academic pressure, and more competing demands on time and energy, all of which may undermine sustained physical activity participation. Existing evidence shows that university students face multiple barriers and facilitators related to physical activity, and that digital health interventions can improve some activity outcomes in this population, although the effects are not always consistent across indicators such as steps, moderate-to-vigorous activity, and sedentary behavior (Bi et al., 2024; Brown et al., 2024).

At the same time, AI-enabled health tools are becoming increasingly relevant in health behavior support. Compared with conventional digital platforms, AI-based systems can provide more adaptive, interactive, and personalized assistance, such as tailored exercise suggestions, conversational feedback, reminders, progress monitoring, and health-data interpretation. Recent reviews have further highlighted the broad functional scope of AI in digital health. AI applications have increasingly been used to support diagnosis, continuous monitoring, personalized intervention, and patient-centered health management, including behavioral and mental health support (Ali et al., 2025). From a capability- and function-oriented perspective, contemporary AI health systems also encompass a range of task-specific functions, including prediction, monitoring, decision support, and personalized healthcare assistance, underscoring that AI health tools should be understood in terms of the functions they provide rather than as a single homogeneous technology category (Abbas et al., 2025). This broader functional perspective is particularly relevant to the present study, in which AI health tools refer to AI-enabled applications, platforms, or device-based systems that provide individualized exercise-related guidance, feedback, monitoring, or recommendation functions. Reviews of AI chatbot interventions have shown that such systems may support behavior change by increasing personalization, immediacy, and accessibility, although their long-term effectiveness still requires further evidence (Aggarwal et al., 2023; Singh et al., 2023; Fu et al., 2026). In the physical activity domain, recent work has also suggested that AI-based agents may function not only as information providers but also as coach-like or companion-like supporters, highlighting the importance of perceived support in digitally assisted exercise contexts (Silacci et al., 2025).

Although interest in AI-supported exercise has grown, the empirical literature remains fragmented. One recent study found that Chinese college students' intention to adopt generative AI for exercise information was positively associated with exercise adherence, and that autonomous motivation mediated this relationship (Gou et al., 2026). Other work has shown that perceived support from fitness apps predicts exercise adherence through self-efficacy and perceived health control (Guan et al., 2026). In addition, AI anthropomorphism has been linked to exercise motivation through technology acceptance and self-efficacy (Wang et al., 2025a), and generative AI health knowledge acquisition has also been associated with greater physical activity partly through exercise self-efficacy (Sun et al., 2025). Together, these findings suggest that AI-related support may matter for exercise behavior, but current evidence has focused more on adoption intention, anthropomorphic features, or generic fitness-app support than on users' perceived exercise support from AI health tools more broadly.

To explain how perceived exercise support from AI health tools may relate to later exercise adherence, the present study draws first on Social Cognitive Theory. According to this perspective, self-efficacy is a central determinant of health behavior because individuals are more likely to initiate and persist in action when they believe they can successfully perform the behavior in question (Bandura, 2004). In the exercise domain, self-efficacy has long been regarded as an important predictor of participation and persistence, and recent evidence continues to support its relevance. A systematic review of ecological momentary assessment studies found that day-level self-efficacy was positively associated with physical activity, indicating that efficacy beliefs are relevant not only across long follow-up periods but also in everyday behavioral regulation (Bittel et al., 2023). Research in Chinese university students likewise links exercise self-efficacy with exercise-related outcomes (Yong and Fan, 2025). These findings provide a strong theoretical basis for expecting supportive AI-assisted exercise experiences to be associated with later confidence in exercising. However, direct prospective evidence testing whether perceived support from AI exercise tools precedes subsequent exercise self-efficacy remains limited.

Self-Determination Theory provides a second and complementary framework. The theory proposes that sustained exercise is more likely when behavioral regulation is autonomous and self-endorsed rather than externally controlled (Deci and Ryan, 2000; Teixeira et al., 2012). Contemporary evidence supports this motivational distinction: a systematic review and multivariate meta-analysis of Self-Determination Theory-based interventions in organized physical activity found positive effects on intrinsic motivation and identified regulation (Manninen et al., 2022). In practical terms, students who feel capable of exercising may be better positioned to internalize exercise goals as personally meaningful and consistent with their own values. Thus, self-efficacy and autonomous motivation may represent adjacent psychological stages rather than isolated predictors. Nevertheless, evidence establishing the temporal ordering of these constructs remains limited in AI-supported exercise contexts.

The proposed serial logic is therefore theoretically plausible, but the recent empirical literature does not yet provide a complete test of it. Emerging studies show that AI-related support, technology characteristics, self-efficacy, motivation, and exercise behavior are associated, but most studies isolate only part of the process or use cross-sectional designs. Three gaps are especially important. First, prospective evidence linking perceived AI exercise support to later self-efficacy is scarce. Second, the temporal relationship between self-efficacy and subsequent autonomous motivation has rarely been examined in AI-supported exercise settings. Third, few studies have integrated perceived AI exercise support, exercise self-efficacy, autonomous motivation, and exercise adherence within a single serial model. These gaps motivate the present three-wave design and the combined use of Social Cognitive Theory and Self-Determination Theory.

Against this background, the present study adopts a three-wave time-lagged design to examine whether perceived exercise support from AI health tools with exercise-support functions predicts later exercise adherence among university students through exercise self-efficacy and autonomous motivation. Specifically, perceived AI exercise support is assessed at T1, exercise self-efficacy at T2, and autonomous motivation together with exercise adherence at T3, while baseline exercise adherence is controlled. By separating the proposed process across time, the present study aims to move beyond prior cross-sectional evidence and provide a more rigorous test of the psychological mechanism through which perceived AI-supported exercise experiences may be translated into sustained exercise adherence among university students.

## 2 Literature review and research hypothesis

### 2.1 AI health tools with exercise-support functions and exercise adherence

For university students, the difficulty of maintaining regular exercise is shaped not only by personal willingness but also by the broader transition to a less structured lifestyle. Earlier work has shown that college students often experience marked declines in regular physical activity after entering university, and that this period is characterized by inconsistent schedules, competing academic demands, and limited behavioral stability (Keating et al., 2005). Qualitative research has further suggested that university students' exercise behavior is strongly influenced by practical constraints, social context, and the extent to which support is perceived as relevant to their actual daily conditions (Deliens et al., 2015). In this sense, sustained exercise adherence depends not merely on intention, but on whether students feel supported in translating exercise plans into manageable routines.

Recent research has also shown that the exercise intention-behavior gap remains a meaningful issue in university populations. Cui (2025) found that college students' physical exercise behavior is influenced by a complex set of psychological and social factors, and that a considerable gap may remain even when exercise intention is already present (Cui, 2025). This suggests that students do not simply need stronger intentions; rather, they need forms of support that help transform intention into persistent behavioral enactment. In this context, AI health tools with exercise-support functions may provide a new type of assistance.

Compared with static digital resources, AI-enabled tools can provide conversational guidance, tailored reminders, feedback based on user input, and more individualized exercise-related suggestions. Earlier systematic reviews have shown that conversational agents in health care are increasingly used to deliver personalized, interactive, and user-responsive support (Laranjo et al., 2018; Kocaballi et al., 2019), and that conversational AI may improve usability, engagement, and support quality in health-related settings (Milne-Ives et al., 2020). More recent evidence extends this understanding to the exercise domain. Wang et al. (2025b) reported in a systematic review and meta-analysis of randomized controlled trials that chatbot-based exercise interventions can improve several exercise-related outcomes. Similarly, Oh et al. (2025) found that a relational artificial intelligence chatbot showed promising feasibility, usability, and initial efficacy for increasing physical activity behavior. In a broader review, Montelaghi et al. (2025) concluded that artificial intelligence-driven virtual assistants for physical activity promotion commonly integrate behavior change techniques such as goal setting, feedback, and motivational support, although the long-term evidence base remains limited. Taken together, these findings suggest that when university students perceive an AI health tool as providing meaningful exercise support, they may be more likely to sustain exercise behavior over time.

*H1: Perceived exercise support from AI health tools at T1 is positively associated with exercise adherence at T3*.

### 2.2 Perceived exercise support from AI health tools and exercise self-efficacy

A key reason why perceived support may matter is that it can shape users' beliefs about their own capability. Research on exercise behavior has long suggested that supportive conditions are unlikely to influence adherence directly unless they also strengthen confidence in one's ability to act consistently. In one influential longitudinal analysis, social support was linked to ongoing exercise behavior partly through efficacy cognitions, suggesting that supportive conditions may increase exercise persistence by changing how capable individuals feel in relation to exercise demands (Duncan and McAuley, 1993). This line of reasoning is especially relevant in environments where exercise is frequently interrupted by time pressure, uncertainty, or low confidence.

Meta-analytic evidence also supports the central role of self-efficacy in exercise-related behavior change. Ashford et al. (2010) showed that intervention strategies designed to strengthen self-efficacy were associated with more favorable physical activity outcomes, and that confidence-building mechanisms are a meaningful target for promoting sustained lifestyle and recreational physical activity (Ashford et al., 2010). In the present context, AI health tools may contribute to such confidence by offering feasible exercise suggestions, adjusting recommendations to users' circumstances, and reducing uncertainty in everyday decision making.

More recent evidence in student populations strengthens this argument. Yu et al. (2024) found that exercise motivation was positively related to college students' self-efficacy, with leisure satisfaction and mental toughness further explaining this association. More directly, Ke et al. (2025) showed that physical self-efficacy served as an important mediator between supportive exercise-related conditions and exercise adherence among college students. Recent micro-longitudinal evidence also indicates that self-efficacy is positively related to day-level physical activity (Bittel et al., 2023). However, these studies primarily concern general exercise environments or non-AI forms of support. Prospective evidence showing that perceived exercise support from AI health tools precedes later exercise self-efficacy remains scarce. Addressing this specific temporal gap is the rationale for testing perceived AI exercise support at T1 against exercise self-efficacy at T2.

*H2: Perceived exercise support from AI health tools at T1 is positively associated with exercise self-efficacy at T2*.

### 2.3 Exercise self-efficacy and autonomous motivation

Although self-efficacy is important, confidence alone may not be sufficient to explain sustained exercise adherence. Self-Determination Theory suggests that long-term exercise behavior is more likely when motivation is autonomous, that is, when behavior is experienced as self-endorsed and personally meaningful rather than externally imposed. A meta-analysis of Self-Determination Theory in health contexts showed that need-supportive environments and competence-related experiences are positively associated with more autonomous forms of motivation and, in turn, with more favorable health behaviors (Ng et al., 2012). This suggests that exercise support may be most effective when it not only helps people feel capable, but also helps them internalize exercise goals (Fortier et al., 2012).

Exercise self-efficacy may play an important role in this internalization process. In the exercise domain, Edmunds et al. (2006) showed that more supportive exercise contexts were linked to stronger need satisfaction and more self-determined motivational regulations (Edmunds et al., 2006). Similarly, Fortier et al. (2007) proposed a self-determination process model in which supportive conditions contribute to physical activity adoption through perceived competence and more self-determined motivation (Fortier et al., 2007). Taken together, these studies suggest that higher exercise self-efficacy may function as an important antecedent of autonomous motivation.

Recent findings are consistent with the proposed link between efficacy beliefs and more internalized motivation. A 2022 systematic review and multivariate meta-analysis showed that Self-Determination Theory-based physical-activity interventions can strengthen intrinsic motivation and identified regulation, reinforcing the importance of competence-supportive experiences for autonomous forms of motivation (Manninen et al., 2022). In college students, Yu et al. (2024) likewise showed that exercise-related psychological processes are closely intertwined, while Ke et al. (2025) found that physical self-efficacy and exercise motivation jointly explained exercise adherence through a serial mechanism. Although exercise motivation is broader than autonomous motivation, these findings support the proposition that stronger efficacy beliefs may be associated with more self-endorsed motivational states. At the same time, direct temporal evidence that exercise self-efficacy precedes later autonomous motivation remains limited, particularly in AI-supported exercise contexts. The T2-to-T3 ordering in the present study was therefore designed to provide a more explicit prospective test of this theoretical step.

*H3: Exercise self-efficacy at T2 is positively associated with autonomous motivation at T3*.

### 2.4 Autonomous motivation, exercise adherence, and the serial mediation pathway

Autonomous motivation is especially relevant to exercise adherence because adherence requires repeated behavioral engagement rather than one-time compliance. Students may begin exercising in response to reminders or short-term external incentives, but such influences are unlikely to sustain adherence unless exercise becomes more internally regulated. Prior work in the exercise domain has consistently shown that more self-determined forms of motivation are associated with stronger and more stable physical activity behavior (Edmunds et al., 2006; Ng et al., 2012). This means that students are more likely to continue exercising when they regard it as personally meaningful rather than merely something they feel they should do.

On this basis, the present study proposes a serial mechanism rather than a set of isolated effects. When university students perceive that AI health tools provide meaningful and individualized exercise support, they may first experience greater confidence in their ability to exercise regularly. That stronger exercise self-efficacy may then facilitate more autonomous motivation, because students who feel capable of acting are more likely to internalize exercise goals and experience exercise as self-endorsed. In turn, stronger autonomous motivation should promote better exercise adherence.

This logic is consistent with earlier support-efficacy models of exercise behavior and with recent college-student evidence (Zhao et al., 2026). Ke et al. (2025) found that physical self-efficacy and exercise motivation exerted a serial-mediating effect on exercise adherence, while recent AI-related studies have independently linked supportive or personalized digital experiences with self-efficacy, motivation, and exercise-related behavior. Nevertheless, few studies have integrated perceived AI exercise support, exercise self-efficacy, autonomous motivation, and exercise adherence within one model that introduces temporal separation across the earlier stages of the process. The present study addresses this gap by assessing perceived AI exercise support at T1, exercise self-efficacy at T2, and autonomous motivation and exercise adherence at T3. Because autonomous motivation and adherence are assessed concurrently at T3, the final component is treated as a within-wave association; accordingly, the proposed serial effect is interpreted as an indirect association consistent with the hypothesized sequence rather than as proof of a fully longitudinal causal mechanism.

*H4: Perceived exercise support from AI health tools at T1 shows a positive serial indirect association with exercise adherence at T3 through exercise self-efficacy at T2 and autonomous motivation at T3, with the final motivation-adherence component interpreted as a within-wave association*.

## 3 Materials and methods

### 3.1 Participants

This study used an observational three-wave survey design and recruited participants from Guangdong Pharmaceutical University and Hunan First Normal University through university-based online channels, including class WeChat groups, student networks, and campus social media platforms. The target population was full-time undergraduate and postgraduate students who were existing users of AI health tools with exercise-support functions. This sampling frame was selected because perceived AI exercise support can be meaningfully evaluated only by students with direct experience of such functions; accordingly, the study was designed to examine associations among existing users rather than to estimate the effect of initiating AI-tool use in the general university-student population.

In the present study, AI health tools with exercise-support functions were defined as digital applications, platforms, or device-based systems that use AI-based conversational, recommendation, or data-analysis functions to provide exercise-related support. Such support could include exercise suggestions, training-plan generation, adaptive reminders, progress feedback, interpretation of exercise- or health-related data, or exercise adjustments based on users' perceived health status. Examples included AI chatbots or generative AI tools used for exercise guidance, AI-enabled fitness or health apps, AI-based virtual exercise coaches, and wearable-device applications that provide AI-generated exercise recommendations or feedback.

Participants were eligible for inclusion if they met all of the following criteria: (1) they were aged between 18 and 26 years; (2) they were full-time university students enrolled at Guangdong Pharmaceutical University or Hunan First Normal University; (3) they had used at least one AI health tool with exercise-support functions within the past 30 days; (4) they had used such a tool to obtain exercise-related support at least three times during the past month, including exercise suggestions, training plans, exercise reminders, interpretation of exercise- or health-related data, or exercise adjustments based on perceived health status; and (5) they had used such tools continuously for at least 1 month rather than on a one-time trial basis. The frequency and duration thresholds were intended to ensure sufficient exposure for participants to evaluate perceived support from the focal tool. They also mean that the analytic population represents established users, a point considered explicitly when interpreting generalizability and potential selection bias.

Participants were excluded if they met any of the following conditions: (1) they were physical education majors or varsity athletes; (2) they reported exercise contraindications, serious sports injuries, or recent postsurgical recovery within the past 3 months; (3) they reported no actual use of exercise-support functions during the past 30 days, even if they had used AI health tools for other purposes such as general health consultation or information search; (4) they failed to complete all three waves of the survey; or (5) their responses showed obvious carelessness, extremely short response times, or could not be matched across waves.

### 3.2 Measures

#### 3.2.1 Perceived AI exercise support

Perceived AI exercise support was assessed at T1. Before completing the perceived AI exercise support items, participants were asked to focus on the primary AI health tool with exercise-support functions that they had identified at T1. Therefore, the phrase “this AI tool” in all items referred specifically to the participant's self-identified primary AI exercise-support tool, rather than to AI health tools in general.

The measure was adapted primarily from the Need Support Scale in Fitness Apps, which includes 12 items covering autonomy support, competence support, and relatedness support. In the present study, references to fitness apps were replaced with AI health tools with exercise-support functions so that the wording matched the target context. Three perceived-fit items were added to capture the personalized and adaptive nature of AI-supported exercise guidance, resulting in a 15-item scale. Example items include “This AI tool provides exercise suggestions that fit my actual situation,” “This AI tool helps me feel supported in maintaining exercise,” and “This AI tool adjusts its recommendations based on my feedback.” All items were rated on a 7-point Likert scale from 1 (strongly disagree) to 7 (strongly agree), and the mean score was used in the main analysis, with higher scores indicating stronger perceived AI exercise support. Importantly, this construct represents the user's perceived supportive experience with the focal tool rather than an objective assessment of the technical quality, clinical accuracy, or effectiveness of the AI system itself.

#### 3.2.2 Exercise self-efficacy

Exercise self-efficacy was measured at T2 using the Chinese version of the Exercise Self-Efficacy Scale. This scale has been used in recent Chinese university student research and consists of 8 items. Participants rated each item on a 5-point Likert scale ranging from 1 strongly disagree to 5 strongly agree, with higher scores indicating stronger confidence in their ability to persist in exercise under various circumstances. Example items assessed confidence in maintaining exercise regularly and confidence in continuing exercise despite barriers (Huang et al., 2025; Yong and Fan, 2025).

#### 3.2.3 Autonomous motivation

Autonomous motivation was measured at T3 using the autonomous motivation component of the Chinese version of the Behavioral Regulation in Exercise Questionnaire-3. Following the psychometric recommendations from the Chinese BREQ-3 validation study, the autonomous motivation score was represented by the combined identified regulation, integrated regulation, and intrinsic motivation items. In the present study, 11 items were retained to represent this construct. Each item was rated on a 5-point scale ranging from 1 not true for me to 5 very true for me, with higher scores indicating stronger autonomous motivation toward exercise (Luo et al., 2022).

#### 3.2.4 Exercise adherence

Exercise adherence was measured at both T1 and T3 using the Exercise Adherence Scale developed by Wang Shen's team and applied in recent Chinese exercise studies. The scale contains 14 items distributed across three dimensions: behavioral habits, effort investment, and emotional experience. All items were rated on a 5-point Likert scale ranging from 1 strongly disagree or not true to 5 strongly agree or very true, with higher total scores indicating stronger exercise adherence. The T1 assessment was used as the baseline control for exercise adherence at T3 (Li et al., 2024).

#### 3.2.5 Background variables and continuity checks

At T1, participants also reported baseline demographic and background information, including sex, academic year, school affiliation, self-rated health, height and weight for BMI calculation, and duration of AI health tool use. To monitor whether the focal tool context remained relevant during follow-up, two continuity-check items were administered at both T2 and T3. These items asked whether the participant had continued to use AI exercise-support functions during the preceding 2 weeks and how frequently such functions had been used. The continuity-check items were used to verify ongoing engagement with AI exercise-support functions during the study period and were not treated as core constructs in the main structural model.

### 3.3 Procedure

Data were collected across three waves over approximately 4 weeks. T1 was administered on Day 0, T2 on Day 14 ± 2 days, and T3 on Day 28 ± 2 days. The design introduced temporal separation across the earlier hypothesized stages: perceived AI exercise support was measured at T1 and exercise self-efficacy at T2, while autonomous motivation and exercise adherence were measured at T3; baseline exercise adherence at T1 was controlled in the structural model. The design should therefore be described as partially time-lagged rather than fully longitudinal for every path. In particular, because autonomous motivation and exercise adherence were assessed concurrently at T3, their association cannot establish temporal direction and was interpreted as a within-wave component of the proposed sequence. Figure 1 illustrates this measurement schedule and analytic framework.

**Figure 1:**
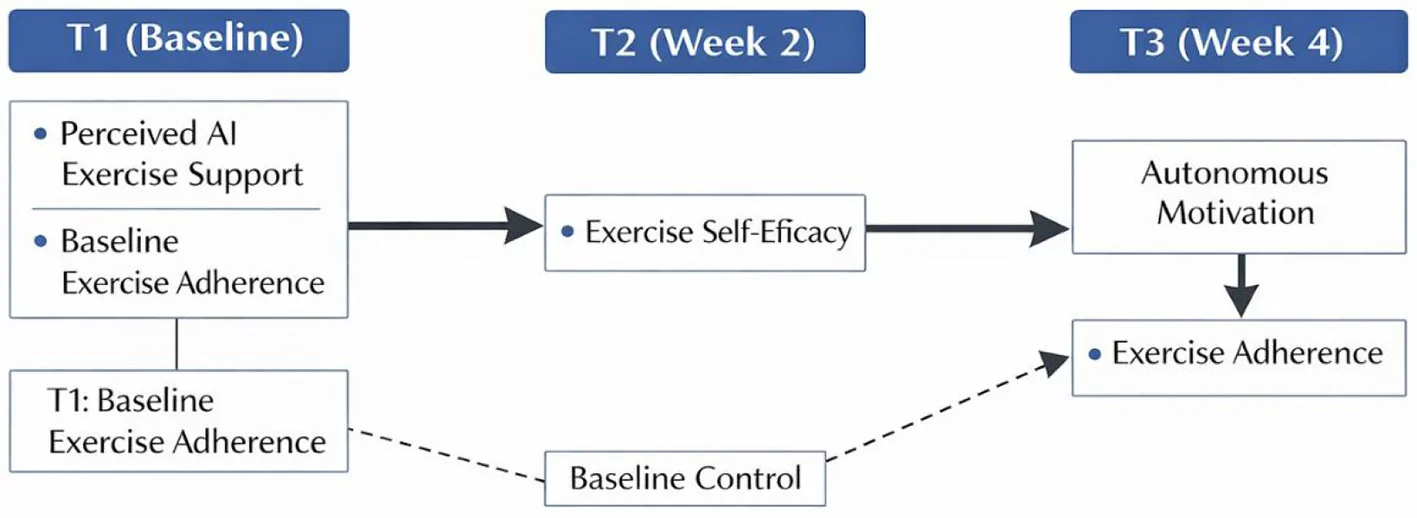
Three-wave time-lagged research framework of the present study.

Participants were invited through university-based online channels such as student groups, class networks, and campus social media platforms. Before beginning T1, participants read an online information sheet and provided electronic informed consent. To enable anonymous matching across the three waves, each participant generated a unique self-matching code based on simple personal prompts that did not reveal direct identifying information.

The T1 questionnaire included the screening items, AI health tool use background, identification of the participant's primary AI exercise-support tool, perceived AI exercise support, baseline exercise adherence, and baseline covariates. Participants who had used more than one AI health tool with exercise-support functions were instructed to select the tool they had used most frequently for exercise-related support during the past 30 days and to answer the perceived support items with reference to that primary tool. T2 assessed exercise self-efficacy and continuity of AI tool use during the preceding 2 weeks. T3 assessed autonomous motivation, exercise adherence, and continuity of AI tool use during the preceding 2 weeks.

Each survey link remained open for 72 h, and a reminder was sent 48 h after the initial invitation. To reduce attrition, participants received a small incentive after completing each wave and an additional completion incentive after finishing all three waves. The estimated completion time was approximately 7 to 9 min at T1, 3 to 4 min at T2, and 5 to 6 min at T3.

### 3.4 Statistical analysis

All statistical analyses were performed using SPSS 27.0 and Mplus 8.7. First, the dataset was screened to remove ineligible responses, incomplete questionnaires, careless responses, and unmatched cases across waves. Descriptive statistics, Pearson correlations, and Cronbach's alpha coefficients were then calculated for the main variables. Attrition analysis was conducted by comparing participants who completed all three waves with those who dropped out after T1 or T2 on the baseline variables. Independent-samples *t tests* were used for continuous variables, and chi-square tests were used for categorical variables.

Second, because the focal variables were assessed using self-report measures, common method bias was examined before the main model testing. Harman's single-factor test was conducted by entering all self-report items from the focal variables into an unrotated exploratory factor analysis.

Third, confirmatory factor analysis was used to evaluate the measurement properties of the main scales. To examine discriminant validity, the hypothesized measurement model was compared with alternative models. Because exercise adherence was measured at both T1 and T3, longitudinal measurement invariance across these two waves was also tested in a sequence of nested models, including configural, metric, and scalar invariance models. Missing data in the longitudinal model were handled using full information maximum likelihood.

Fourth, the hypothesized serial mediation model was tested using structural equation modeling. The structural model specified paths from T1 perceived AI exercise support to T2 exercise self-efficacy, from T2 exercise self-efficacy to T3 autonomous motivation, and from T3 autonomous motivation to T3 exercise adherence. T1 exercise adherence was included as a baseline control when predicting T3 exercise adherence. The direct path from T1 perceived AI exercise support to T3 exercise adherence was retained to test whether the mediation pattern was partial or full. Sex, academic year, BMI, self-rated health, school affiliation, and duration of AI tool use were entered as covariates. Because autonomous motivation and exercise adherence were both assessed at T3, the final link in the serial pathway reflected an association estimated within the final wave after temporal separation of the earlier stages and control of baseline exercise adherence.

Finally, indirect effects were estimated using bias-corrected bootstrap procedures with 5,000 resamples. The total effect, direct effect, total indirect effect, and hypothesized serial indirect effect were examined, and an indirect effect was considered statistically significant when the 95% confidence interval did not include zero. Because autonomous motivation and exercise adherence were both measured at T3, the serial indirect effect was interpreted as a model-based indirect association consistent with the proposed g7 ordering of constructs, not as evidence that the complete mediation chain was temporally or causally established.

## 4 Results

### 4.1 Sample characteristics and attrition analysis

A total of 684 students completed the baseline survey at T1. At T2, 556 participants returned the second-wave questionnaire, and at T3, 487 participants completed the final-wave questionnaire. After excluding cases that did not meet the eligibility criteria, provided incomplete responses, failed the attention-check items, or could not be successfully matched across the three waves, the final analytic sample consisted of 438 participants, representing a retention rate of 64.0% relative to the T1 sample.

The final sample was drawn from Guangdong Pharmaceutical University and Hunan First Normal University. Of the retained participants, 228 (52.1%) were from Guangdong Pharmaceutical University and 210 (47.9%) were from Hunan First Normal University. The mean age of the final sample was 20.74 years (SD = 1.89). In total, 286 participants (65.3%) were female and 152 (34.7%) were male. The mean body mass index (BMI) was 21.58 (SD = 3.14), and the mean duration of AI health tool use was 5.37 months (SD = 3.28). Additional sample characteristics are presented in Table 1.

**Table 1:** Sample characteristics of the final sample and attrition analysis.

Variable	Final matched sample (*N* = 438)	Dropout sample (*N* = 246)	Test statistic	*p*
Age, M (SD)	20.74 (1.89)	20.55 (1.96)	*t* = 1.21	0.228
BMI, M (SD)	21.58 (3.14)	21.37 (3.26)	*t* = 0.86	0.390
Perceived AI exercise support (T1), M (SD)	4.96 (0.88)	4.84 (0.91)	*t* = 1.35	0.177
Exercise adherence (T1), M (SD)	3.41 (0.67)	3.33 (0.71)	*t* = 1.02	0.309
Female, *n* (%)	286 (65.3%)	151 (61.4%)	*χ^2^* = 0.95	0.329
Male, *n* (%)	152 (34.7%)	95 (38.6%)	—	—
Guangdong Pharmaceutical University, *n* (%)	228 (52.1%)	132 (53.7%)	*χ^2^* = 0.41	0.521
Hunan First Normal University, *n* (%)	210 (47.9%)	114 (46.3%)	—	—
Undergraduate, *n* (%)	372 (84.9%)	216 (87.8%)	*χ^2^* = 1.34	0.247
Postgraduate, *n* (%)	66 (15.1%)	30 (12.2%)	—	—

The dropout sample refers to participants who completed T1 but did not complete all three waves. BMI, Body Mass Index. Independent-samples *t tests* were used for continuous variables, and chi-square tests were used for categorical variables.

To examine potential attrition bias, participants who completed all three waves were compared with those who dropped out after T1 or T2 on baseline variables. Independent-samples *t tests* and chi-square tests showed no significant differences in age, BMI, perceived AI exercise support at T1, baseline exercise adherence, sex, academic year, or school affiliation (all ps >0.05). These findings suggest that attrition was unlikely to have introduced substantial systematic bias into the analytic sample.

### 4.2 Continuity of AI tool use during follow-up

To verify that the focal tool context remained relevant during the follow-up period, the continuity-check items assessed at T2 and T3 were examined. Among the final analytic sample, 397 participants (90.6%) at T2 reported that they had continued to use AI exercise-support functions during the preceding 2 weeks, and the corresponding number at T3 was 374 (85.4%). The reported frequency of AI exercise-support function use remained at a moderate-to-high level across the follow-up period, although a slight decline was observed from T2 to T3. These findings suggest that most participants remained engaged with AI exercise-support functions during the study period.

### 4.3 Common method bias test

Because the focal constructs were assessed using self-report measures, common method bias was examined before the main analyses. Harman's single-factor test extracted nine factors with eigenvalues greater than 1, and the first unrotated factor accounted for 28.47% of the total variance. This proportion was below the commonly used threshold, suggesting that common method bias was unlikely to substantially affect the interpretation of the findings.

### 4.4 Measurement model evaluation and longitudinal invariance testing

Before testing the structural model, confirmatory factor analysis was conducted to evaluate the measurement properties of the focal constructs. The hypothesized five-factor model, including perceived AI exercise support at T1, exercise self-efficacy at T2, autonomous motivation at T3, exercise adherence at T1, and exercise adherence at T3, showed an acceptable fit to the data, χ^2^/df = 2.31, CFI = 0.935, TLI = 0.928, RMSEA = 0.055, and SRMR = 0.049. All standardized factor loadings were significant and ranged from 0.62 to 0.89, indicating adequate representation of the latent constructs.

Alternative models that combined conceptually distinct variables showed poorer fit than the hypothesized five-factor model, supporting discriminant validity among the focal constructs (Table 2). Because exercise adherence was assessed at both T1 and T3, longitudinal measurement invariance was further tested. The configural, metric, and scalar invariance models all showed acceptable fit, and the changes in CFI and RMSEA across nested models were small (Table 3). These findings support the longitudinal comparability of the Exercise Adherence Scale across the two time points.

**Table 2:** Comparison of the measurement models.

Model	*χ^2^*/df	CFI	TLI	RMSEA	SRMR
Five-factor model	2.31	0.935	0.928	0.055	0.049
Four-factor model (self-efficacy + autonomous motivation combined)	3.08	0.901	0.892	0.069	0.061
Three-factor model (support + self-efficacy +motivation combined)	3.74	0.872	0.861	0.079	0.073
One-factor model	6.21	0.721	0.694	0.109	0.112

The five-factor model included perceived AI exercise support (T1), exercise self-efficacy (T2), autonomous motivation (T3), exercise adherence (T1), and exercise adherence (T3).

**Table 3:** Longitudinal measurement invariance testing for exercise adherence across T1 and T3.

Model	*χ^2^*/df	CFI	TLI	RMSEA	SRMR	ΔCFI	ΔRMSEA
Configural invariance	2.41	0.948	0.939	0.057	0.046	—	—
Metric invariance	2.32	0.946	0.940	0.055	0.049	0.002	0.002
Scalar invariance	2.28	0.943	0.940	0.054	0.051	0.003	0.001

T1 = baseline; T3 = week 4. Changes in CFI and RMSEA across nested models were within acceptable ranges, supporting longitudinal measurement invariance.

### 4.5 Preliminary relationships and structural model testing

#### 4.5.1 Descriptive statistics and correlation matrix

Table 4 presents the means, standard deviations, Cronbach's alpha coefficients, and Pearson correlation coefficients for the main study variables. The pattern of bivariate associations is further visualized in Figure 2. Perceived AI exercise support at T1 was positively correlated with exercise self-efficacy at T2, autonomous motivation at T3, exercise adherence at T1, and exercise adherence at T3 (rs = 0.31 to.43, ps < 0.001). Exercise self-efficacy at T2 was positively correlated with autonomous motivation at T3 and exercise adherence at T3 (rs = 0.46 to.48, ps < 0.001). Autonomous motivation at T3 was also positively correlated with exercise adherence at T3 (*r* = 0.52, *p* < 0.001). In addition, baseline exercise adherence at T1 was positively correlated with exercise adherence at T3 (*r* = 0.58, *p* < 0.001), indicating temporal stability in exercise adherence across the study period. Cronbach's alpha coefficients ranged from 0.89 to 0.94, indicating satisfactory internal consistency for all focal variables. Overall, the pattern of correlations was consistent with the hypothesized model.

**Table 4:** Means, standard deviations, reliabilities, and correlations among the main study variables.

Variable	*M*	SD	1	2	3	4	5
1. Perceived AI exercise support (T1)	4.96	0.88	0.94				
2. Exercise self-efficacy (T2)	3.72	0.64	0.43^***^	0.91			
3. Autonomous motivation (T3)	3.81	0.67	0.34^***^	0.48^***^	0.92		
4. Exercise adherence (T1)	3.41	0.67	0.31^***^	0.29^***^	0.33^***^	0.89	
5. Exercise adherence (T3)	3.57	0.65	0.36^***^	0.46^***^	0.52^***^	0.58^***^	0.90

Values on the diagonal are Cronbach's α coefficients. T1 = baseline; T2 = week 2; T3 = week 4. ^***^ *p* < 0.001.

**Figure 2:**
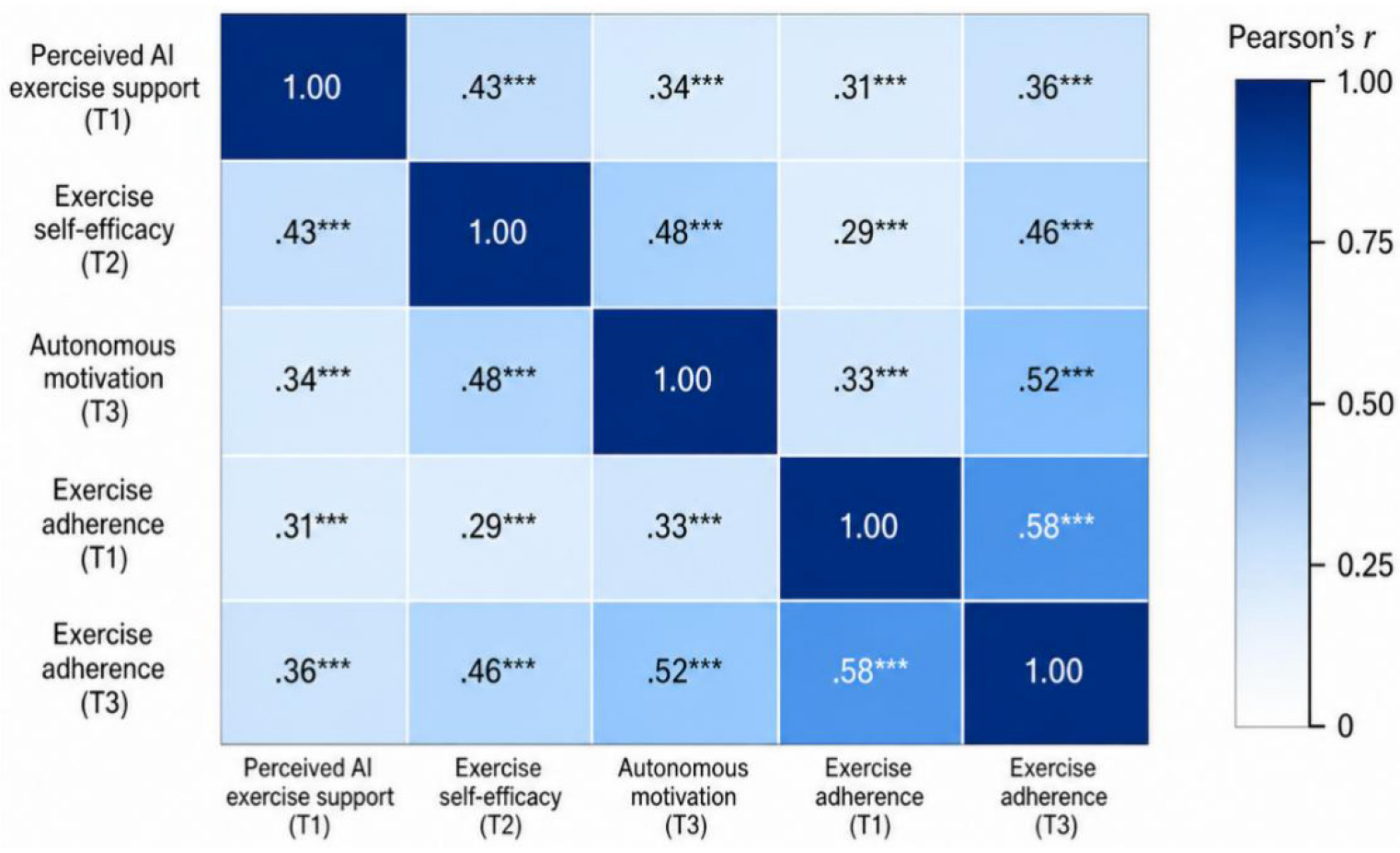
Correlation heatmap of the main study variables. Cell values represent Pearson correlation coefficients among the main study variables. Diagonal values represent self-correlations. Darker colors indicate stronger positive correlations. All off-diagonal correlations were significant at *p* < 0.001. T1 = baseline; T2 = week 2; T3 = week 4.

#### 4.5.2 Structural model fit and hypothesis testing

After the measurement model had been established, the hypothesized structural model was tested using structural equation modeling. The model showed an acceptable fit to the data, χ^2^/df = 2.18, CFI = 0.941, TLI = 0.934, RMSEA = 0.052, and SRM*r* = 0.047. The standardized path coefficients of the final structural model are presented in Figure 3, and the detailed parameter estimates are reported in Table 5. Perceived AI exercise support at T1 positively predicted exercise self-efficacy at T2 (β = 0.36, *p* < 0.001), supporting H2. Exercise self-efficacy at T2 positively predicted autonomous motivation at T3 (β =.041, *p* < 0.001), supporting H3. Autonomous motivation at T3 was positively associated with exercise adherence at T3 (β = 0.29, *p* < 0.001), consistent with the proposed serial pathway. After baseline exercise adherence and covariates were controlled, the direct path from perceived AI exercise support at T1 to exercise adherence at T3 remained significant (β = 0.11, *p* = 0.028). Baseline exercise adherence at T1 also significantly predicted exercise adherence at T3 (β = 0.47, *p* < 0.001), indicating substantial temporal stability across the study period. Among the covariates, only self-rated health showed a significant positive association with exercise adherence at T3 (β = 0.09, *p* = 0.037), whereas sex, academic year, BMI, school affiliation, and duration of AI tool use were not significant (all ps >0.05).

**Figure 3:**
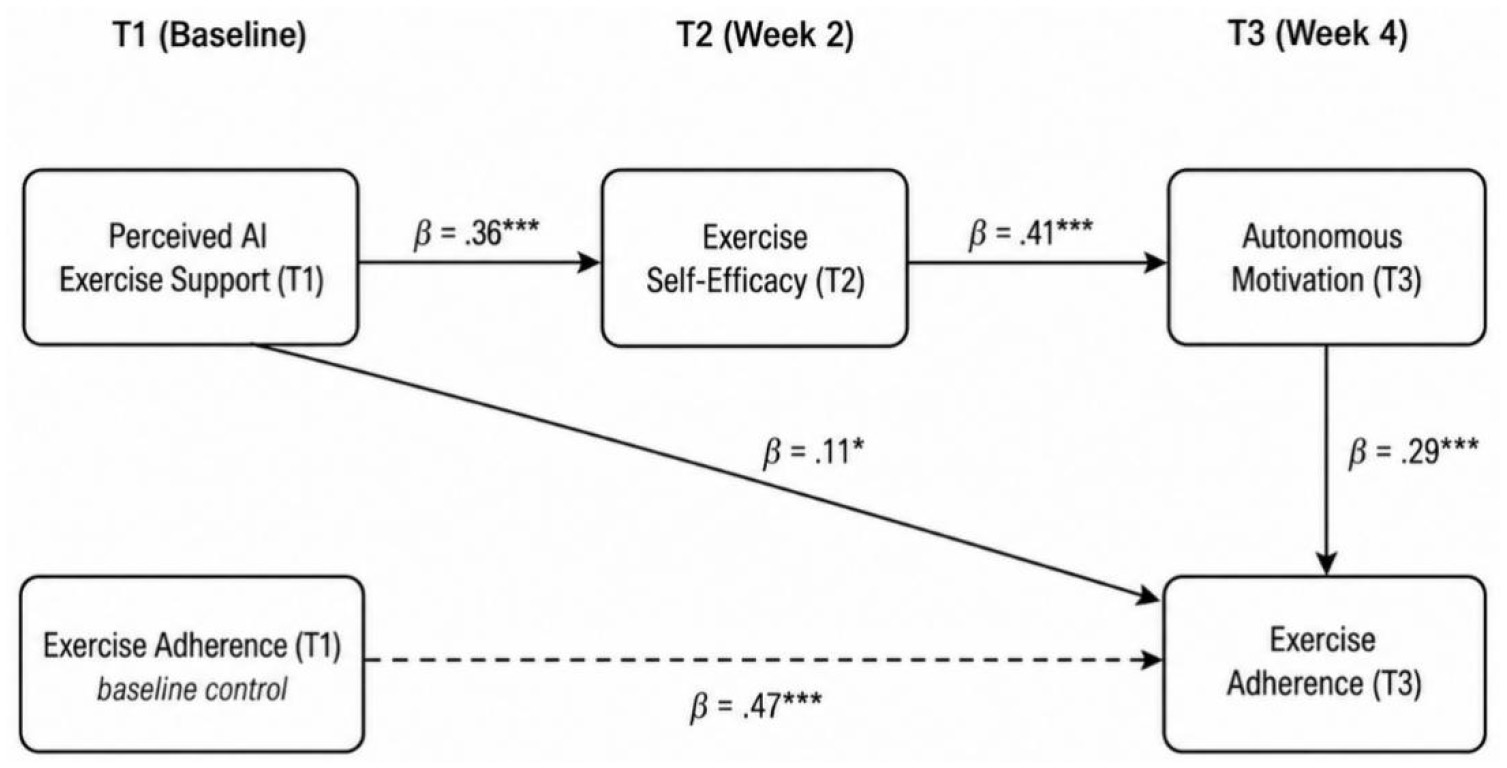
Standardized path coefficients of the serial mediation model. Note. Standardized coefficients are shown. T1 = baseline; T2 = week 2; T3 = week 4. Baseline exercise adherence and covariates were controlled. Covariate paths are omitted for clarity. * *p* < 0.05, *** *p* < 0.001.

**Table 5:** Structural path coefficients of the hypothesized model.

Path	Standardized coefficient (β)	SE	*p*
T1 Perceived AI exercise support → T2 Exercise self-efficacy	0.36	0.05	< 0.001
T2 Exercise self-efficacy → T3 Autonomous motivation	0.41	0.06	< 0.001
T3 Autonomous motivation → T3 Exercise adherence	0.29	0.05	< 0.001
T1 Perceived AI exercise support → T3 Exercise adherence	0.11	0.05	0.028
T1 Exercise adherence → T3 Exercise adherence	0.47	0.05	< .001
Sex → T3 Exercise adherence	−0.04	0.03	0.214
Academic yea*r* → T3 Exercise adherence	−0.03	0.02	0.287
BMI → T3 Exercise adherence	−0.05	0.03	0.148
Self-rated health → T3 Exercise adherence	0.09	0.04	0.037
School affiliation → T3 Exercise adherence	−0.02	0.03	0.421
Duration of AI tool use → T3 Exercise adherence	0.06	0.03	0.091

Sex was coded as 0 = male and 1 = female. School affiliation was coded as 0 = Guangdong Pharmaceutical University and 1 = Hunan First Normal University. BMI = body mass index. Standardized coefficients are reported.

### 4.6 Serial mediation effects

Bias-corrected bootstrap analyses with 5,000 resamples were conducted to test the total, direct, and indirect effects of perceived AI exercise support at T1 on exercise adherence at T3. As shown in Table 6, the total effect was significant (β = 0.15, 95% CI [0.05, 0.25], *p* =0.004), supporting H1. The total indirect effect was also significant (β = 0.04, 95% CI [0.02, 0.07]). More importantly, the hypothesized serial indirect effect from perceived AI exercise support at T1 to exercise adherence at T3 through exercise self-efficacy at T2 and autonomous motivation at T3 was significant (β =0.04, 95% CI [0.02, 0.06]). Because the confidence interval did not include zero, H4 was supported. After the mediators and baseline exercise adherence were included in the model, the direct effect remained significant (β = 0.11, 95% CI [0.01, 0.21], *p* = 0.028), indicating that the association between perceived AI exercise support and later exercise adherence was partially rather than fully mediated by the proposed serial pathway.

**Table 6:** Bootstrap estimates of total, direct, and indirect effects of perceived AI exercise support on exercise adherence.

Effect	Standardized effect (β)	Boot SE	95%CI	*p*
Total effect	0.15	0.05	[0.05, 0.25]	0.004
Direct effect	0.11	0.05	[0.01, 0.21]	0.028
Total indirect effect	0.04	0.01	[0.02, 0.07]	—
Serial indirect effect (T1 support → T2 self-efficacy → T3 autonomous motivation → T3 adherence)	0.04	0.01	[0.02, 0.06]	—

Bootstrap confidence intervals were based on 5,000 bias-corrected resamples. An indirect effect was considered statistically significant when the 95% confidence interval did not include zero.

## 5 Discussion

The present study examined whether perceived exercise support from AI health tools with exercise-support functions was associated with later exercise adherence among university students, and whether this association was linked to exercise self-efficacy and autonomous motivation. Several findings deserve emphasis. First, perceived AI exercise support at T1 was positively associated with exercise adherence at T3, even after baseline exercise adherence and relevant covariates were controlled. Second, perceived AI exercise support at T1 was positively associated with exercise self-efficacy at T2, and exercise self-efficacy at T2 was positively associated with autonomous motivation at T3. Third, the hypothesized sequential indirect association from perceived AI exercise support to later exercise adherence through exercise self-efficacy and autonomous motivation was significant, whereas the direct association remained significant, indicating partial rather than full mediation. In addition, baseline exercise adherence showed substantial temporal stability, and most participants continued to use AI exercise-support functions during the follow-up period, suggesting that the focal behavioral context remained relevant across waves. Together, these findings suggest that the role of AI health tools in exercise behavior may not be limited to providing reminders or information. Rather, when such tools are perceived as genuinely supportive, they may be linked to sustained exercise adherence through a psychological process involving greater confidence in one's ability to exercise and stronger internalization of exercise goals. In this sense, the present findings support a psychologically meaningful pathway linking perceived AI-supported exercise experiences to later exercise maintenance among university students.

The positive association between perceived AI exercise support and later exercise adherence is consistent with the view that regular exercise in university settings depends not only on intention, but also on whether support is experienced as practical, personalized, and usable in everyday life. University students often face unstable routines, academic pressure, and competing demands on time and energy. Under such conditions, AI health tools may help narrow the intention–behavior gap by translating vague exercise intentions into more manageable actions. Tailored exercise suggestions, adaptive reminders, progress feedback, and ongoing interaction may reduce the burden of planning and sustaining exercise, thereby increasing the likelihood that students remain engaged over time. This finding also extends recent literature on AI-supported exercise. Previous work has linked the intention to adopt generative AI for exercise information to exercise adherence, and other studies have shown that perceived support from fitness apps may influence adherence through psychological mechanisms. The present study goes beyond these lines of work by focusing specifically on perceived support from AI health tools with exercise-support functions and by testing its association with later exercise adherence in a three-wave design. The persistence of a direct association after accounting for the proposed mediators suggests that perceived AI exercise support may have a role that is not fully reducible to the two psychological variables examined here. This implies that other mechanisms, such as habit support, perceived accountability, enjoyment, perceived responsiveness of the tool, or perceived health control, may also contribute to the relationship between AI-supported exercise experiences and later adherence.

A central finding of the present study is that the observed associations were consistent with the proposed sequential pathway involving exercise self-efficacy and autonomous motivation between perceived AI exercise support and later exercise adherence. This pattern is theoretically important because it suggests that perceived support may be associated with later behavior through a staged psychological process rather than through a single-step pathway. Specifically, students who perceived stronger exercise support from AI health tools reported greater exercise self-efficacy 2 weeks later, and this higher self-efficacy was associated with stronger autonomous motivation at the final wave, which in turn was concurrently associated with better exercise adherence. The first stage of this pathway is well aligned with Social Cognitive Theory. Self-efficacy reflects the belief that one can successfully perform a behavior under real-world conditions, and this belief is especially important when the behavior requires persistence in the face of barriers. In the present context, AI health tools may be linked to higher exercise self-efficacy by making exercise more concrete, more achievable, and less uncertain. For example, when students receive suggestions that fit their actual situation, reminders that match their schedule, and adaptive feedback that responds to their progress, they may become more confident that regular exercise is feasible. This interpretation is consistent with the present finding that perceived AI exercise support was associated with later exercise self-efficacy and with prior exercise research highlighting the central role of efficacy beliefs in adherence-related behavior.

The second stage of the pathway is well explained by Self-Determination Theory. Although confidence is important, sustained exercise behavior is less likely to be maintained if it remains externally prompted and is not personally endorsed. Students who feel capable of exercising may be more likely to internalize exercise goals, experience exercise as congruent with their own values, and regulate their behavior in a more autonomous manner. In this study, exercise self-efficacy at T2 was positively associated with autonomous motivation at T3, and autonomous motivation at T3 was in turn positively associated with exercise adherence at T3. This pattern suggests that confidence may create the psychological conditions under which exercise becomes more self-endorsed, and this more internalized motivational state may help sustain repeated behavioral engagement. Importantly, the mediation pattern was partial rather than full. The sequential indirect association was significant, but the direct association from perceived AI exercise support to later exercise adherence remained significant after the mediators were entered. This indicates that exercise self-efficacy and autonomous motivation explain an important part of the relationship, but not all of it. Accordingly, the present model should be understood as a meaningful but incomplete account of how AI-supported exercise experiences may be linked to behavioral maintenance.

The present findings have several theoretical implications. First, they directly address the gap between broad theoretical predictions and the fragmented recent empirical literature by integrating perceived AI exercise support, exercise self-efficacy, autonomous motivation, and exercise adherence within a single model. Social Cognitive Theory provides a basis for the T1 support-to-T2 self-efficacy association, whereas Self-Determination Theory helps explain why greater perceived competence may be linked to more autonomous regulation of exercise. Recent evidence supporting day-level associations between self-efficacy and physical activity (Bittel et al., 2023) and meta-analytic evidence on Self-Determination Theory-based motivational interventions (Manninen et al., 2022) further support the relevance of these mechanisms. Second, the findings move the AI-exercise literature beyond technology adoption alone by focusing on perceived support as a psychologically meaningful characteristic of users' experiences. Third, the model suggests that the two theoretical frameworks may capture adjacent stages of the same broader process rather than competing explanations. This contribution should nevertheless be interpreted as a partial temporal test: the earlier support-to-efficacy-to-motivation ordering is separated across waves, whereas the final motivation-adherence association remains within T3.

The findings also offer practical implications for developers, universities, and campus health services. For developers of AI health tools, the results suggest that effective exercise-support functions should do more than deliver generic reminders. Tools may be more beneficial when they help users feel capable of exercising and support their sense of autonomy. In practical terms, this means that features such as tailored exercise recommendations, adaptive goal setting, progress visualization, barrier-specific feedback, and flexible interaction styles may be especially valuable. For universities, the results suggest that AI-assisted exercise support may be a promising supplement to campus physical activity promotion. Rather than promoting tool use in a general sense, institutions may gain more by encouraging forms of AI use that help students feel more capable of exercising and more personally invested in maintaining exercise. Another practical point concerns behavioral continuity. Baseline exercise adherence remained a strong correlate of later adherence, indicating that exercise behavior already shows meaningful temporal stability even within a relatively short follow-up window. This suggests that early support may be particularly important when students are still in the process of forming exercise routines. In addition, most participants reported continued use of AI exercise-support functions during follow-up, which lends practical relevance to the behavioral context captured in the present study.

The present study has several strengths. It adopted a three-wave design with partial temporal separation, controlled for baseline exercise adherence, examined a theoretically grounded sequential mediation pathway, and focused on an emerging but still underexplored context, namely perceived exercise support from AI health tools with exercise-support functions among university students. These design features strengthen the contribution of the study relative to prior cross-sectional work and provide a more informative basis for interpreting the temporal ordering of the earlier stages of the proposed mechanism. At the same time, several limitations should be acknowledged. First, although the design introduced temporal separation between perceived AI exercise support, exercise self-efficacy, and the final-wave variables, autonomous motivation and exercise adherence were both measured at T3. Accordingly, the final motivation–adherence link should be interpreted with caution, because this part of the model does not provide full temporal separation. Second, all focal variables were assessed through self-report measures. Although common method bias did not appear to be severe, the use of self-report data may still introduce shared-method variance and social desirability influences. Third, the sample was drawn from two universities in China and included only students who had already used AI health tools with exercise-support functions for at least 1 month. This criterion was used to ensure that participants had sufficient experience to meaningfully evaluate the perceived exercise support provided by these tools. However, it may also have introduced selection bias, because students who discontinued use early or found such tools unhelpful were less likely to be represented. This may have restricted the range of perceived AI exercise support and potentially affected the magnitude of the observed support–adherence association. Therefore, the findings should primarily be generalized to existing users of AI exercise-support tools rather than to university students in general. Future studies should include nonusers, new users, and users who discontinue AI tools to further examine these relationships. Fourth, the study focused on perceived support from AI health tools in general and did not fully distinguish between specific types of AI tools or exercise-support functions. Different tools, such as generative AI chatbots, AI-enabled fitness apps, AI-based virtual coaches, and wearable-device applications, may provide different forms of support. Fifth, although the direct and indirect associations were statistically significant, the observed associations should not be interpreted as establishing strong causal claims. Additional longitudinal studies with longer follow-up periods, experimental designs, and more objective behavioral indicators are still needed.

Future research could extend the present work in several directions. One useful step would be to adopt fuller longitudinal separation by assessing autonomous motivation and exercise adherence at different later time points. This would allow future studies to test the final motivation–adherence link more rigorously. Another direction would be to incorporate behavioral trace data, wearable-device indicators, or system logs to complement self-report measures of exercise adherence and AI tool use. It would also be valuable to examine other mechanisms that may operate alongside self-efficacy and autonomous motivation, such as enjoyment, perceived accountability, habit formation, perceived health control, trust in AI recommendations, or anthropomorphic perceptions of AI support. Finally, future studies could compare different types of AI health tools and different exercise-support features in order to clarify which forms of AI support are most conducive to sustained physical activity in university populations. More broadly, future studies could situate AI-supported exercise within the wider digital health intervention landscape. Recent evidence indicates that digital health interventions, including mobile health applications, wearable technologies, and AI-based platforms, may contribute to health promotion and behavioral adherence, while their effectiveness is also shaped by broader implementation and policy contexts (Faizan et al., 2025). Accordingly, future research could examine not only the psychological mechanisms of AI-supported exercise, but also how institutional support, accessibility, and implementation strategies influence sustained engagement with these tools.

## 6 Conclusion

In conclusion, the present study provides partially time-lagged evidence that, among university students who already use AI health tools for exercise support, stronger perceived AI exercise support is associated with later exercise adherence. The observed pattern is consistent with a sequential psychological process involving exercise self-efficacy and autonomous motivation: perceived support at T1 was associated with self-efficacy at T2, which was associated with autonomous motivation at T3, while autonomous motivation was concurrently associated with exercise adherence at T3. Because the final two constructs were measured at the same wave, this last component should be interpreted as a within-wave association rather than as evidence of a fully longitudinal causal mediation pathway. Overall, the findings suggest that supportive, personalized, and autonomy-relevant experiences with AI exercise tools may be psychologically meaningful for existing users, while stronger causal conclusions will require designs with fuller temporal separation and experimental or objective behavioral evidence.

## Data Availability

The raw data supporting the conclusions of this article will be made available by the authors, without undue reservation.
